# Retrograde and oscillatory shear increase across the menopause transition

**DOI:** 10.14814/phy2.13965

**Published:** 2019-01-03

**Authors:** Yasina B. Somani, David J. Moore, Danielle Jin‐Kwang Kim, Joaquin U. Gonzales, Matthew A. Barlow, Steriani Elavsky, David N. Proctor

**Affiliations:** ^1^ Department of Kinesiology Penn State University University Park Pennsylvania; ^2^ Penn State Harrisburg Middletown Pennsylvania; ^3^ Penn State College of Medicine Hershey Pennsylvania; ^4^ Texas Tech University Lubbock Texas; ^5^ Eastern New Mexico University Portales New Mexico; ^6^ University of Ostrava Ostrava Czech Republic

**Keywords:** Menopause transition, oscillatory shear, retrograde shear

## Abstract

Declines in endothelial function can take place rapidly across the menopause transition, placing women at heightened risk for atherosclerosis. Disturbed patterns of conduit artery shear, characterized by greater oscillatory and retrograde shear, are associated with endothelial dysfunction but have yet to be described across menopause. Healthy women, who were not on hormone therapy or contraceptives, were classified into early perimenopausal, late perimenopausal, and early postmenopausal stage. Resting antegrade, retrograde, and oscillatory shear were calculated from blood velocity and diameter measured in the brachial and common femoral artery using Doppler ultrasound. Serum was collected for measurements of estradiol, follicle‐stimulating hormone (FSH), and luteinizing hormone. After adjusting for age, brachial artery oscillatory shear was significantly higher in early postmenopausal women (*n* = 15, 0.17 ± 0.08 a.u.) than both early (*n* = 12, 0.08 ± 0.05 a.u., *P* < 0.05) and late (*n* = 8, 0.08 ± 0.04 a.u) perimenopausal women, and retrograde shear was significantly greater in early postmenopausal versus early perimenopausal women (−19.47 ± 12.97 vs. −9.62 ± 6.11 sec^−1^, both *P* < 0.05). Femoral artery oscillatory and retrograde shear were greater, respectively, in early postmenopausal women (*n* = 15, 0.19 ± 0.08 a.u.; −13.57 ± 5.82 sec^−1^) than early perimenopausal women (*n* = 14, 0.11 ± 0.08 a.u.; −8.13 ± 4.43 sec^−1^, *P* < 0.05). Further, Pearson correlation analyses revealed significant associations between FSH and both retrograde and oscillatory shear, respectively, in the brachial (*r* = −0.40, *P* = 0.03; *r* = 0.43, *P* = 0.02) and common femoral artery (*r* = −0.45, *P* = 0.01; *r* = 0.56, *P* = 0.001). These results suggest menopause, and its associated changes in reproductive hormones, adversely influences conduit arterial shear rate patterns to greater oscillatory and retrograde shear rates.

## Introduction

Patterns of arterial shear stress, the frictional force exerted by blood flow, are integral in the maintenance of vascular health (Malek et al. 1999). Specifically, high steady laminar flow induces intracellular calcium release stimulating endothelial release of vasodilators that inhibit blood coagulation, smooth muscle proliferation, and adhesion of molecules to the endothelial surface (Traub and Berk 1998). Conversely, highly oscillatory flow that includes a large retrograde (backward) component fails to increase intracellular calcium transients (Helmlinger et al. 1995), thus providing a weak stimulus for endothelial release of vasoactive substances (Ziegler et al. 1998). Indeed, experimental evidence in humans demonstrates that elevations in retrograde shear have a dose‐dependent effect on decreasing endothelium‐dependent dilation as measured via brachial artery flow‐mediated dilation (FMD) in young men (Thijssen et al. 2009). Brachial artery FMD represents a surrogate, noninvasive measure of nitric oxide (NO)‐dependent endothelium‐mediated vasodilator function that independently predicts future cardiovascular events in healthy individuals, beyond traditional cardiovascular disease (CVD) risk factors (Inaba et al. 2010; Shechter et al. 2014). Further, reductions in NO bioavailability, a hallmark of aging that is caused by both a reduction in synthesis and increased inactivation of NO, are associated with impairments to brachial artery FMD (Taddei et al. 2001; Seals et al. 2006). The inverse association found between retrograde shear and brachial artery FMD in young men may be extended to women. Women who develop preeclampsia have greater resting brachial artery retrograde shear after pregnancy as compared to age‐matched healthy controls (Scholten et al. 2014). The elevated retrograde shear in these preeclamptic women is also associated with lower brachial artery FMD (Scholten et al. 2014).

Aging is associated with increases in retrograde and oscillatory shear in the brachial (Credeur et al. 2009; Padilla et al. 2011; Casey et al. 2012) and femoral arteries (Young et al. 2010). Padilla and colleagues showed that NO synthase inhibition in the forearm circulation of young participants increased retrograde and oscillatory shear to values that were similar to those observed in older participants (Padilla et al. 2011). This finding suggests that changes in conduit artery shear in older adults are associated with reduced NO bioavailability. Age‐associated increases in sympathetic outflow to vascular beds (Dinenno and Joyner 2006) also likely contributes to altered shear patterns in older adults since acute elevation in muscle sympathetic nerve activity increase retrograde and oscillatory shear in younger participants (Padilla et al. 2010; Casey et al. 2012). While much information is available for the effect of chronological aging on retrograde and oscillatory shear, the effect of reproductive aging, specifically menopause in women, on shear patterns has not yet been investigated.

The menopause transition in women is a period marked by an accelerated rise in CVD risk factors, which coincides with a time where there are profound changes to the reproductive hormone milieu (Matthews et al. 2009; Novella et al. 2012). The Study of Women's Health Across the Nation (SWAN) was a large prospective, longitudinal trial that identified the adverse changes in CVD risk factors that take place in aging women. While some risk factors tended to follow a linear model, suggestive of chronological aging, others followed a non‐linear pattern that was suggestive of hormonal influences. Specifically, a precipitous rise in total cholesterol, low density lipoprotein (LDL) cholesterol, and apolipoprotein B took place within 1 year of the final menstrual period, and these changes were not consistent with the linear model of chronological aging (Matthews et al. 2009). Endothelium‐dependent vasodilation has an apparent decline that is more rapid in women across menopause. Moreau and colleagues demonstrated in a cross‐sectional analysis that brachial artery FMD was progressively reduced with menopausal stage (Moreau et al. 2012). This decline was associated with reduced estradiol in women and was independent of chronological age as well as other CVD risk factors (Moreau et al. 2012). While changes to endothelial function have been established in women across the menopause transition (Moreau and Hildreth 2014), changes in the pattern of conduit artery shear stress have not been described and may represent a contributing factor to endothelial dysfunction in postmenopausal women.

The purpose of the present cross‐sectional study was, therefore, to assess resting patterns of conduit artery shear in women at discrete stages of the menopause transition. We hypothesized that both resting retrograde and oscillatory shear in the brachial and femoral artery would progressively increase in magnitude with menopausal stage. We further hypothesized that increases in retrograde and oscillatory shear would associate with changes in reproductive hormones across the menopause transition.

## Methods

### Participants

Healthy women between the age of 40–60 years were recruited from Centre County, Pennsylvania. Using the Stages of Reproductive Aging Workshop (STRAW) criteria, based on self‐reported bleeding, women were classified into early perimenopausal, late perimenopausal, and early postmenopausal stages (Soules et al. 2001). Specifically, women were designated early perimenopausal if they reported variable cycle length (>7 days different from normal), and late perimenopausal if they reported two or more skipped cycles or ≥60 days of amenorrhea (but less than 12 months). Early postmenopausal women reported not experiencing a menstrual cycle during the previous 12 months but were less than 5 years past their final menstrual period (Soules et al. 2001).

All participants had a resting blood pressure below 140/90 mmHg based on brachial blood pressure (HEM‐705CP, Omron) measured following 5 min of seated rest, taken by a trained nurse. Women included in the present study were healthy as determined by a medical history questionnaire provided by the Penn State General Clinical Research Center, a physical examination, a resting electrocardiogram, and plasma lipid concentrations. Participants with extremes of body mass index (BMI <18 or >40 kg/m^2^) computed from height and weight measurements were disqualified from the study. Participants included in the study were not taking hormone therapy, contraceptives, herbal supplements, antihypertensive, or lipid‐lowering medications at the time of study or within 6 months of participating. Participants were asked to refrain from food and caffeine ingestion for 12 h prior to experimental visits, as well as to refrain from vigorous exercise and alcohol intake for 24 h prior. Participants were provided written consent to participate in the study after receiving an explanation of the experimental procedures and possible risks associated with participation. This study was approved by the Office for Research Protections at The Pennsylvania State University in agreement with the guidelines set forth by the Declaration of Helsinki (IRB #: 29153).

### Reproductive hormones

Concentrations of reproductive hormones were measured from 10 h fasted blood samples and, in perimenopausal women with monthly cycles, between days 3–5 of menstrual bleeding. Blood samples were allowed to clot for 30 min at room temperature and then were centrifuged at 1006 ***g*** for 15 min at 4°C.

Estradiol‐17*β* was analyzed using liquid chromatography tandem mass spectrometry. Analytical sensitivity for the estradiol assay was 2 pg·mL^−1^. Follicle‐stimulating hormone (FSH) and luteinizing hormone (LH) were analyzed using an immunochemiluminometric assay with analytical sensitivity of 0.07 and 0.7 MIU·mL^−1^ for FSH and LH, respectively. Reproductive hormones were unable to be measured in five women (two women for estradiol and three women for FSH). All samples were analyzed by Quest Diagnostics.

### BMI and body composition

Participants had their height (m) and weight (kg) measured by a trained nurse in the Penn State Clinical Research Center to determine BMI (kg·m^−2^). While participants were supine, dual X‐ray absorptiometry (model QDR 4500W, Hologic, Waltham, MA) was used to measure whole body composition. Total body fat and fat‐free mass (FFM) were measured with standard cut lines. Abdominal adiposity was calculated from manually defined specific regions of interest, specifically from the upper edge of the first lumbar vertebra to the anterior superior iliac spine.

### Cardiorespiratory fitness testing

Each participant performed a modified Balke treadmill test to maximal effort on a separate day from experimental visits. The test was graded and involved a 2 min warm‐up at 2.5 miles per hour followed by adjustment of speed to elicit 70–75% of age‐predicted maximal heart rate (208−0.7 × age; Tanaka et al. 2001) after which point the speed remained the same throughout the exercise test. The intensity of exercise increased every 2 min by increasing the elevation by 2.5% until the participant reached volitional fatigue. Pulmonary oxygen uptake was measured using indirect calorimetry (TrueOne 2400, Parvomedics, Sandy, Utah) and VO_2peak_ was determined as the highest 15 sec average. All women achieved a peak respiratory exchange ratio (RER)>1.0. To minimize the potential effects of habitual endurance exercise training on brachial and femoral artery shear patterns, only women who were below the 75th percentile of normative cardiorespiratory values for their age group were included in this analysis (Kaminsky et al. 2015).

### Resting conduit artery shear patterns

**Figure 1:**
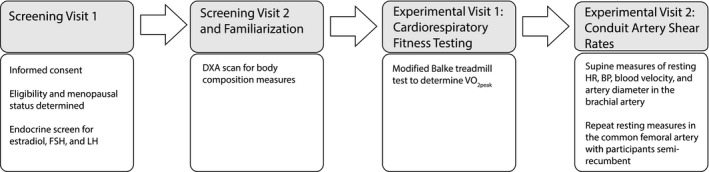
Study timeline. FSH, follicle‐stimulating hormone; LH, luteinizing hormone; DXA, dual energy X‐ray absorptiometry; HR, heart rate; BP, blood pressure.

Diameter and blood flow velocity of the brachial artery and common femoral artery were measured using Doppler ultrasound using a 12 MHz linear‐array probe (HDI 5000, Philips, Bothell, Washington). Resting brachial artery measures were collected with participants supine, while femoral artery measurements were collected with participants in a semirecumbent position. The latter semirecumbent position was used to allow participants to perform knee kick exercise more comfortably for outcome variables that are not reported in this study. Velocity measurements were sampled in real time (400 Hz) using a data acquisition system (Powerlab, AD Instruments, Castle Hill, Australia). Mean blood velocity was calculated from a 60 sec resting period and Doppler signals were collected at a 60° insonation angle. High‐resolution diameter measurements were recorded directly to DVD during rest and were obtained with the artery insonated perpendicularly. Brachial artery and femoral artery diameter were measured across the cardiac cycle using edge‐detection software (Brachial Analyzer Software, Medical Imaging Applications, Iowa City, Iowa). Lastly, mean arterial blood pressure was measured continuously during the resting blood velocity measurement using a beat‐by‐beat finger cuff arterial blood pressure system (Finometer MIDI, Finapress Medical Systems, Netherlands). Please see Figure 1. for the study timeline.

### Data analysis

Baseline recordings of brachial and common femoral artery mean diameter and velocity were used to calculate mean, antegrade, and retrograde shear rate. Mean blood velocities in the forward direction during systole (antegrade) and in the backward direction during diastole (retrograde) were determined with customized functions on Power Lab's chart application (Lab Chart v 8.1.12, AD Instruments, Colorado Springs, US), and shear rate (sec^−1^) was defined as 8*velocity/diameter (Parker et al. 2009). Antegrade and retrograde shear rates were calculated from time‐averaged antegrade and retrograde blood velocities, respectively. Oscillatory shear index (OSI), an indicator of the magnitude of shear oscillation, was defined as: (|retrograde shear|)/ (|antegrade shear| + |retrograde shear|). OSI values, expressed in arbitrary units (a.u.), range from 0 to 0.5. A value of 0 represents unidirectional shear, while 0.5 represents oscillation with time‐average shear equal to 0. Mean artery blood flow was calculated based on continuous measurement of blood velocity and resting artery diameter, and was determined by multiplying the cross‐sectional area (*πr* ^2^) of the artery with resting mean blood velocity. Vascular resistance was calculated by dividing mean arterial blood pressure by mean blood flow.

Statistical analyses were performed using SPSS 25.0 (SPSS, Chicago, IL) software. All data were reported as means ± SD unless stated otherwise and statistical significance was assumed at *P* < 0.05. One‐way ANOVAs were used to examine differences between group characteristics across menopausal stage. To adjust for the influence of age, ANCOVA's were employed to assess differences in resting conduit artery shear rates and hemodynamics. Bonferroni post‐hoc tests were conducted when significant differences were detected. Pearson's correlation coefficient was used to examine the relation between retrograde and oscillatory shear in the brachial artery and femoral artery with reproductive hormones (LH, FSH, and estradiol), cardiorespiratory fitness, age, BMI, and vascular resistance. To assess reproductive hormones as a predictor of arterial shear, a multiple regression analysis was employed to statistically control for the influence of covariates.

## Results

Of the 43 women who met the study criteria, eight were excluded from brachial artery analysis and six from common femoral artery analysis due to low quality resting blood velocity measurements. Of the total 37 women who participated in vascular assessments, 30 women had measures that were included in both brachial artery and femoral artery analysis. Subject characteristics are presented in Tables 1 and 2 for brachial artery and common femoral artery groups, respectively. Early postmenopausal women were older than both early and late perimenopausal women in both groups and had lower cardiorespiratory fitness in the brachial artery group (*P* < 0.05). There were no differences in height, weight, and BMI between the three menopausal stages in both groups. With respect to reproductive hormones, plasma concentrations of FSH and LH were significantly lower, and estradiol was higher in early perimenopausal women than in late perimenopausal and early postmenopausal women (*P* < 0.05). Resting systolic and diastolic blood pressure were not significantly different across menopausal stage.

**Table 1:** Subject characteristics: brachial artery group

Brachial artery (*n* = 35)	Early perimenopausal	Late perimenopausal	Early postmenopausal
*n*	12	8	15
Age (years)	49 ± 2	49 ± 2	55 ± 3*, †
Height (cm)	162 ± 6	164 ± 5	161 ± 12
Weight (kg)	63 ± 6	64 ± 5	70 ± 12
Body composition			
BMI (kg·m^−2^)	24.2 ± 2.7	23.7 ± 1.9	25.7 ± 3.3
Body fat (%)	31.4 ± 5.9	32 ± 3.8	36.3 ± 4.6*
Abdominal adipose (kg)	1.6 ± 0.6	1.8 ± 0.3	2.2 ± 1.0
Cardiorespiratory fitness			
VO_2peak_ (mL·kg^−1^·min^−1^)	31.8 ± 3.9	31. 8 ± 4.2	26.2 ± 4.5*, †
VO_2peak_ (mL·kg^−1^·min^−1^ FFM^−1^)	48.7 ± 4.3	49.2 ± 4.8	43.9 ± 4.7*, †
Reproductive hormones			
FSH (mIU·mL^−1^)	11.1 ± 7.3	71.7 ± 21.1*	78.6 ± 20.5*
Estradiol (pg·mL^−1^)	153 ± 213	24 ± 8*	24 ± 12*
LH (mIU·mL^−1^)	10 ± 8	42 ± 14*	42 ± 17*
Blood pressure			
Resting systolic BP (mmHg)	114 ± 9	112 ± 8	120 ± 11
Resting diastolic BP (mmHg)	76 ± 6	74 ± 7	78 ± 7

Values are mean ± SD. BMI, body mass index; VO_2peak_, peak oxygen uptake; FFM, fat‐free mass; FSH, follicle‐stimulating hormone; LH, luteinizing hormone; BP, blood pressure.

* Significantly different from early perimenopausal (*P* ≤ 0.05).

† Significantly different from late perimenopausal (*P* ≤ 0.05).

**Table 2:** Subject characteristics: common femoral artery group

Femoral Artery (*n* = 37)	Early perimenopausal	Late perimenopausal	Early postmenopausal
*n*	14	8	15
Age (years)	49 ± 3	49 ± 2	55 ± 2*, †
Height (cm)	163 ± 6	164 ± 5	163 ± 13
Weight (kg)	65 ± 7	64 ± 5	70 ± 13
Body composition			
BMI (kg·m^−2^)	24.4 ± 2.8	23.7 ± 1.9	24.6 ± 2.3
Body fat (%)	30.9 ± 6.5	32 ± 3.9	35.7 ± 4.6
Abdominal adipose (kg)	1.6 ± 0.8	1.8 ± 0.3	2.1 ± 1.1
Cardiorespiratory fitness			
VO_2peak_ (mL·kg^−1^·min^−1^)	31.5 ± 5.3	31. 8 ± 4.2	28.0 ± 4.9
VO_2peak_ (mL·kg^−1^·min^−1^ FFM^−1^)	48.5 ± 4.3	49.3 ± 4.8	45.1 ± 5.0
Reproductive hormones			
FSH (mIU/mL)	12.9 ± 8.4	71.7 ± 21.1*	77.7 ± 20.3*
Estradiol (pg·mL^−1^)	173 ± 228	24 ± 8*	25 ± 11*, †
LH (mIU·mL^−1^)	11 ± 8	42 ± 14*	44 ± 17*
Blood pressure			
Resting systolic BP (mmHg)	116 ± 9	112 ± 8	114 ± 14
Resting diastolic BP (mmHg)	76 ± 5	74 ± 7	81 ± 15

Values are mean ± SD. BMI, body mass index; VO_2peak_, peak oxygen uptake; FFM, fat‐free mass; FSH, follicle‐stimulating hormone; LH, luteinizing hormone; BP, blood pressure.

* Significantly different from early perimenopausal (*P* ≤ 0.05).

† Significantly different from late perimenopausal (*P* ≤ 0.05).

### Conduit arterial shear rates

**Figure 2:**
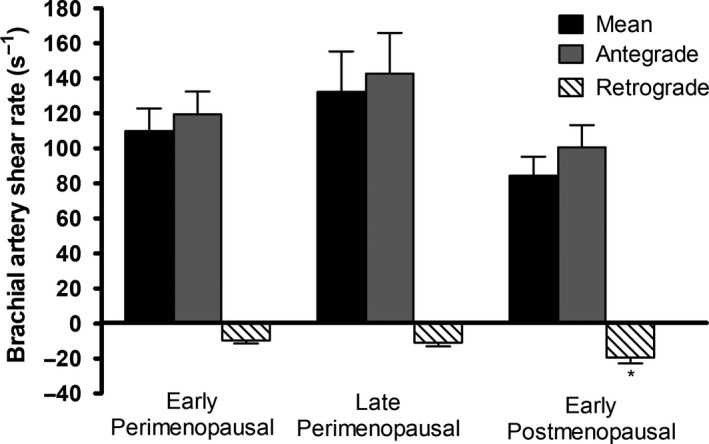
Brachial artery mean, antegrade, and retrograde shear in early perimenopausal (*n* = 12), late perimenopausal (*n* = 8), and early postmenopausal women (*n* = 15). *Significantly different from early perimenopausal (*P* < 0.05, ±SEM).

**Figure 3:**
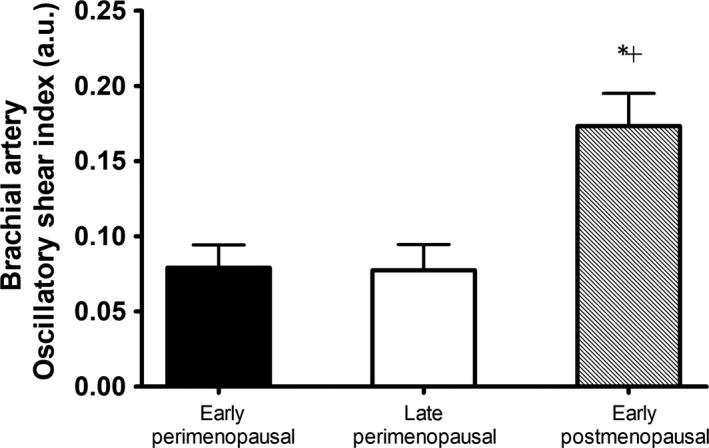
Brachial artery oscillatory shear index (OSI) in early perimenopausal (*n* = 12), late perimenopausal (*n* = 8), and early postmenopausal women (*n* = 15). *Significantly different from early perimenopausal, ^+^Significantly different from late perimenopausal (*P* < 0.05,  ± SEM).

**Figure 4:**
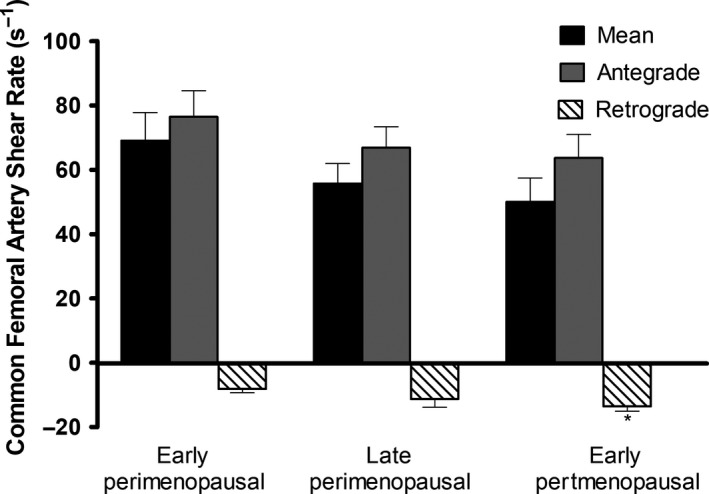
Common femoral artery mean, antegrade, and retrograde shear in early perimenopausal (*n* = 14), late perimenopausal (*n* = 8), and early postmenopausal women (*n* = 15). *Significantly different from early perimenopausal (*P* < 0.05,  ± SEM).

**Figure 5:**
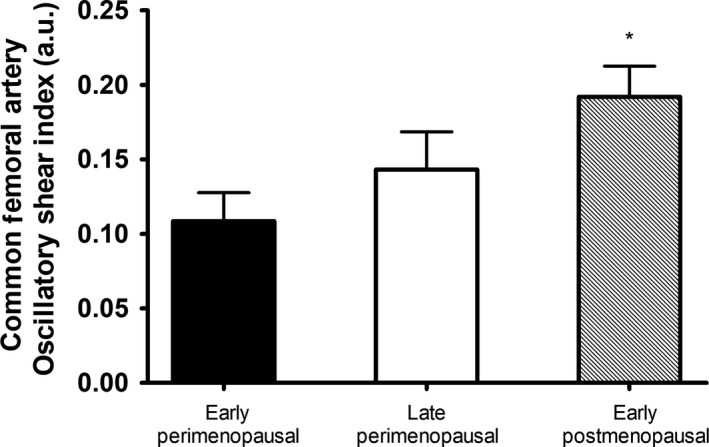
Common femoral artery oscillatory shear index (OSI) in early perimenopausal (*n* = 14), late perimenopausal (*n* = 8), and early postmenopausal women (*n* = 15). *Significantly different from early perimenopausal (*P* < 0.05,  ± SEM).

No significant differences in brachial artery and common femoral artery diameter, mean blood velocity, or mean blood flow were found in women across menopausal stage (Table 3). Mean shear and antegrade shear in both the brachial and femoral artery were not statistically different across menopausal stage (Figs. 2 and 4). Brachial artery retrograde shear was significantly greater in early postmenopausal versus early perimenopausal women (*P* = 0.03), and oscillatory shear was significantly higher in early postmenopausal women than both early (*P* = 0.03) and late (*P* = 0.04) perimenopausal women (Fig. 3). Common femoral artery retrograde shear (Fig. 4) and oscillatory shear (Fig. 5) were greater, respectively, in early postmenopausal women than early perimenopausal women (*P* = 0.01). Observed power for retrograde and oscillatory shear were 0.70 and 0.95 in the brachial artery, and 0.62 and 0.73 in the common femoral artery, respectively.

**Table 3:** Brachial artery and common femoral artery resting hemodynamics

	Early perimenopausal	Late perimenopausal	Early postmenopausal
Brachial artery (*n* = 35)			
Diameter (cm)	0.35 ± 0.04	0.32 ± 0.03	0.35 ± 0.04
Mean blood velocity (cm·sec^−1^)	4.7 ± 1.8	5.1 ± 2.2	3.8 ± 1.88
Mean blood flow (mL·min^−1^)	27 ± 11	24.1 ± 9.5	23.5 ±13.1
MAP (mmHg)	89 ± 6	87 ± 7	92 ± 7
Vascular resistance (mmHg·mL^−1 ^min^−1^)	3.6 ± 1.0	4.1 ± 1.4	5.3 ± 3.9
Femoral artery (*n* = 37)			
Diameter (cm)	0.84 ± 0.07	0.77 ± 0.11	0.85 ± 0.12
Mean blood velocity (cm·sec^−1^)	7.2 ± 3.4	5.3 ± 1.7	5.1 ± 2.6
Mean blood flow (mL·min^−1^)	245.8 ± 123	152.0 ± 70	170.4 ± 86
MAP (mmHg)	93 ± 9	86 ± 11	92 ± 11
Vascular resistance (mmHg·mL^−1 ^min^−1^)	0.55 ± 0.32	0.62 ± 0.25	0.55 ± 0.28

Values are mean ± SD. MAP, mean arterial pressure.

**Figure 6:**
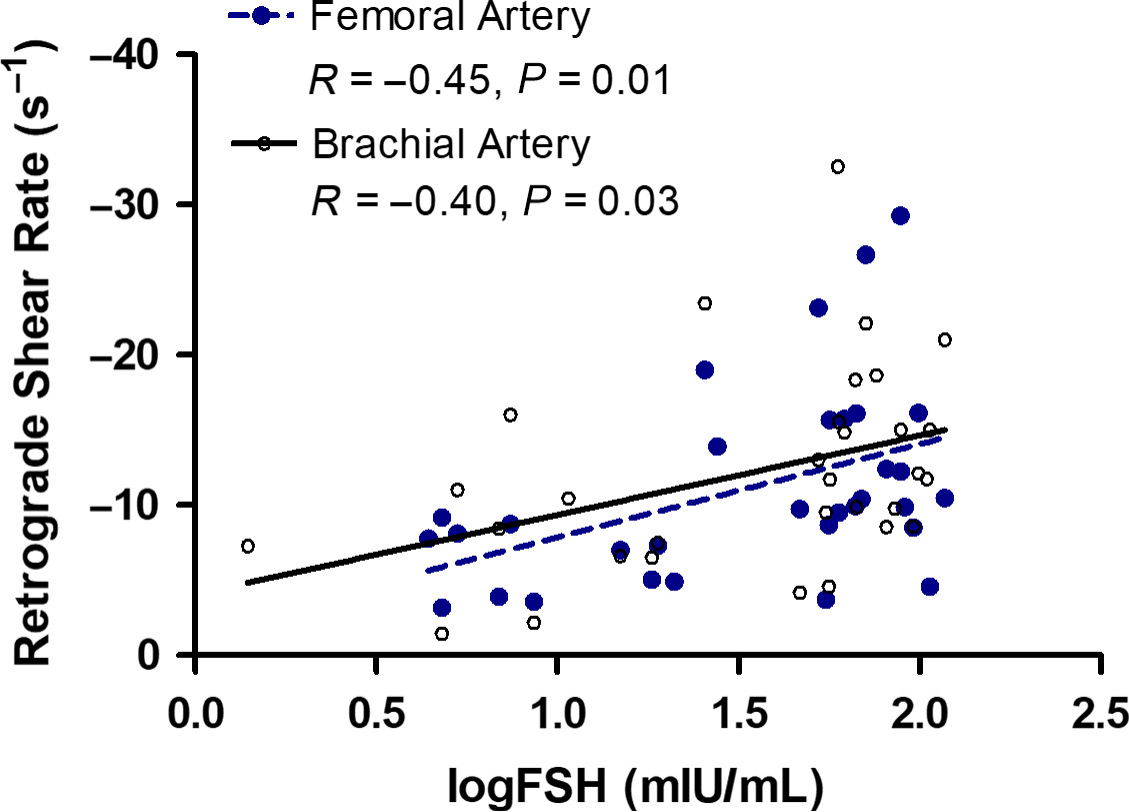
Pearson correlation analysis of FSH and femoral artery (blue) retrograde shear rate (*n* = 32, *r* = −0.45, *P* = 0.01), and brachial artery (black) retrograde shear (*n* = 31, *r* = −0.40, *P* = 0.03).

**Figure 7:**
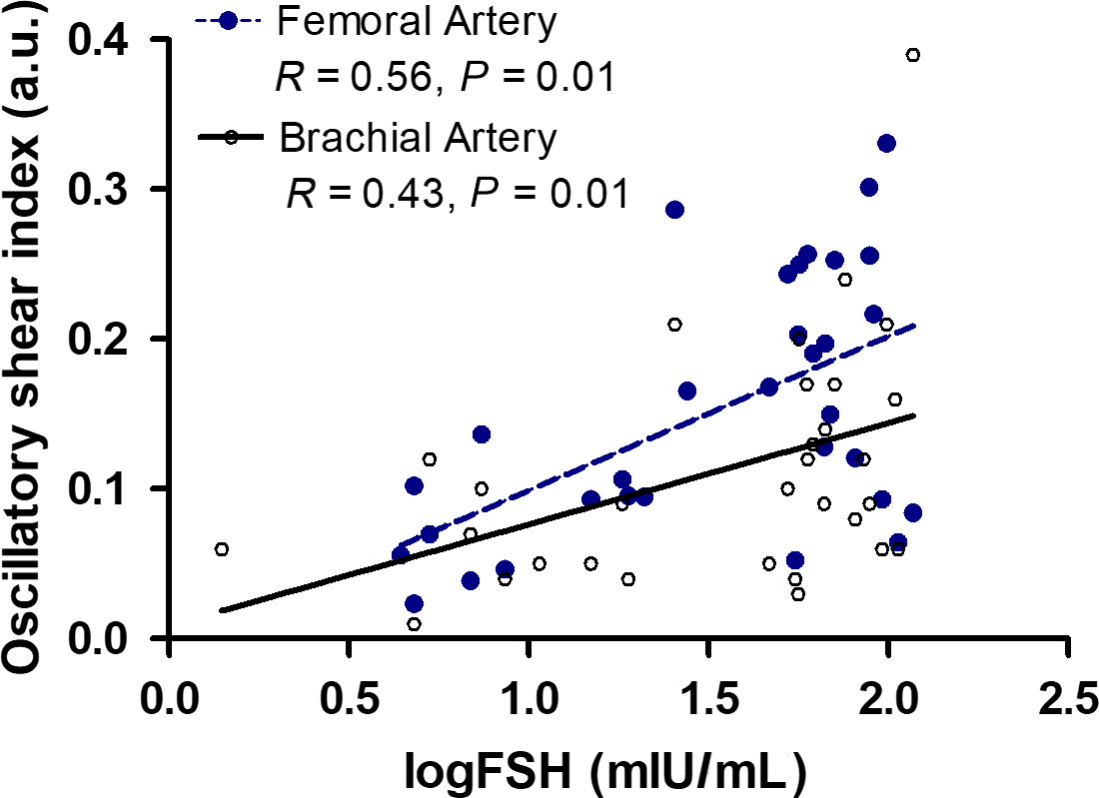
Pearson correlation analysis of FSH and femoral artery (blue) oscillatory shear index (OSI,* n* = 32, *r* = 0.56, *P* = 0.01), and brachial artery (black) OSI (*n* = 31, *r* = 0.43, *P* = 0.01).

In the combined sample of peri‐ and postmenopausal women, significant correlations were found between both retrograde and oscillatory shear in the brachial artery, respectively, with age (*r* = −0.41; *r* = 0.47, *P* < 0.05), VO_2peak_ (*r* = 0.49; *r* = −0.47, *P* < 0.05), VO_2peak_ normalized to FFM (*r* = 0.39; *r* = −0.43, *P* < 0.05), body fat (*r* = −0.48; *r* = 0.43, *P* < 0.05), abdominal adipose (*r* = −0.40; *r* = 0.34, *P* < 0.05), and logFSH (Figs. 6 and 7). When statistically controlling for age, cardiorespiratory fitness, and body composition measures, the associations between both retrograde and oscillatory shear in the brachial artery with logFSH remained significant (*r* = −0.57; *r* = 0.61, *P* < 0.05).

In the common femoral artery, correlation analyses of retrograde and oscillatory shear with age (*r* = −0.35; *r* = 0.50, *P* < 0.05) and logFSH (Figs. 6 and 7) in the combined sample revealed significant associations. After controlling for age, the associations between both femoral artery retrograde and oscillatory shear with FSH, respectively, were still present (*r* = −0.48; *r* = 0.6, *P* < 0.05).

No significant differences in vascular resistance were observed across menopausal stage in either limb group, and no association was found between brachial artery vascular resistance and both retrograde and oscillatory shear, and reproductive hormones (*P* > 0.05). However, significant associations were found between femoral artery vascular resistance and femoral artery retrograde and oscillatory shear (*r* = −0.48; *r* = 0.69, *P* < 0.05), as well as with logFSH (*r* = 0.42, *P* < 0.05). Vascular resistance was not associated with age, body composition measures, or cardiorespiratory fitness in both groups (*P* > 0.05).

## Discussion

Previous studies that have assessed resting conduit arterial shear patterns have primarily focused on the effect of chronological aging. To our knowledge, this is the first study to examine resting conduit arterial shear in both the brachial and common femoral artery in women at discrete stages of the menopause transition. This cross‐sectional investigation found retrograde and oscillatory shear to be higher in postmenopausal women as compared to perimenopausal women in both the brachial and common femoral artery. Furthermore, we showed that blood levels of the reproductive hormone, FSH, to be associated with retrograde and oscillatory shear values independent of age and other confounding factors including body composition measures and cardiorespiratory fitness. These results are intriguing as they provide evidence that reproductive aging has an impact on hemodynamic changes known to contribute to the progression of atherosclerosis.

One possible mechanism by which retrograde and oscillatory shear is elevated in postmenopausal women compared to perimenopausal women may be elevated sympathetic vasoconstriction of downstream arterioles. Padilla and colleagues showed that acute increases to vascular resistance, evoked by elevating sympathetic nervous system activity with graded lower body negative pressure (LBNP), resulted in increased retrograde shear in young men (Padilla et al. 2010). In a following investigation performed in young and old participants, it was found that sympathetic activation via LBNP increased retrograde and oscillatory shear in only young adults to values that were similar to older adults (Casey et al. 2012). Upon alpha‐adrenergic blockade, both retrograde and oscillatory shear in young adults were abolished and in older participants were reduced. While an investigation of this sort has yet to be performed in menopausal women, postmenopausal women are reported to have elevated muscle sympathetic activity (Hart et al. 2012) and elevated overnight cortisol release that is associated with plasma levels of catecholamines and FSH (Woods et al. 2009). It is possible these factors promote greater norepinephrine‐induced vasoconstriction in postmenopausal women resulting in greater retrograde and oscillatory flow patterns in conduit arteries. We assessed vascular resistance but did not find it to differ significantly across menopausal stage in either artery. However, greater vascular resistance was found to correlate with higher levels of retrograde and oscillatory shear in the femoral artery when menopausal women were combined (data not shown). Based on this information, we believe studies assessing muscle sympathetic nervous system activity (MSNA) and conduit artery shear patterns in women across the menopause transition are warranted.

Another possible explanation underlying the observed increase in retrograde and oscillatory shear in early postmenopausal women is reduced NO bioavailability. The association between retrograde shear and NO bioavailability was elucidated by Padilla and colleagues in the forearm circulation of old and young participants (Padilla et al. 2011). Their findings suggest that reduced NO bioavailability in the resistance vessels contributes, in part, to age‐related increases to retrograde and oscillatory shear. Whether this association exists in women across the menopause transition has yet to be explored and warrants investigation. It was previously shown by Moreau and colleagues that brachial artery FMD is progressively reduced in women with menopausal stage, independent of age and other CVD risk factors (Moreau et al. 2012). These authors speculate that reduced estrogen‐mediated generation of NO likely underlies the progressive decline in endothelium‐dependent vasodilation. Findings from a recent meta‐analysis by Green and colleagues suggest that the traditionally used FMD response of conduit arteries to assess endothelial function in humans exhibits substantial NO dependency (Green et al. 2014). As such, strategies to boost NO bioavailability during this narrow period of time may be helpful in lowering the contribution of retrograde and oscillatory shear and slow down accelerated vascular aging in women undergoing menopause.

The finding that FSH was associated with both retrograde and oscillatory shear in both limbs, even after controlling for age and other confounding variables, suggests that reproductive aging in women can drive changes in conduit artery shear patterns. It is possible that the accelerated rise in FSH across the menopause transition may have independent adverse effects on the vasculature (Zhu et al. 2018). A recent study demonstrated that administration of FSH increased atherosclerotic lesions and serum concentrations of vascular cell adhesion molecule‐1 (VCAM‐1) in ovariectomized ApoE knockout mice, which was independent of estrogen deficiency. These authors also found that FSH upregulated VCAM‐1 expression in their cell model of cultured human umbilical vein endothelial cells, and subsequently increased human monocyte adhesion (Li et al. 2017). As such the precipitous increase in FSH, that is associated with reproductive aging during the menopause transition, may independently contribute to the changes we observed in both retrograde and oscillatory shear via direct effects on the vasculature.

Several limitations accompany this study. We found no association between estradiol and resting arterial shear stress. This may be attributed to our experimental methods. Menstrual phase was controlled while sampling blood for hormone levels in perimenopausal women, but we did not control for menstrual cycle phase during experimental visits when vascular assessments took place. Early investigations suggest that menstrual phase can impact vascular measurements, such that brachial artery FMD is lower in the early follicular phase when women are acutely deficient in estrogen than in the late follicular and luteal phase when estrogen is high (Hashimoto et al. 1995; Williams et al. 2001). However, a recent study by Shenouda et al. (2018) demonstrates that changes in estradiol across a natural menstrual cycle are not associated with changes in relative brachial artery FMD. The disparate findings in the literature suggest that mechanisms including but not limited to estrogen receptor function and NO synthase activation may play an important role in keeping endothelial function stable despite fluctuations in estradiol across the menstrual cycle. As discussed earlier, in addition to the loss in estradiol associated with menopause, the rise in FSH that takes place during the menopause transition may also contribute to lowering endothelial function.

To minimize the impact of cardiorespiratory fitness level on shear patterns (Casey et al. 2016) we only included women who were below the 75th percentile of normative cardiorespiratory fitness for their age group according to reference standards (Kaminsky et al. 2015). Despite this attempt, we still found VO_2peak_ to inversely associate with retrograde and oscillatory shear in the brachial artery, thus highlighting the importance of habitual physical activity and exercise in maintaining vascular health. Further, we recognize that early postmenopausal women were older, had higher body fat, and lower cardiorespiratory fitness than early and late perimenopausal women and this represents a limitation to the present study. However, when controlling for these variables the association between FSH and both retrograde and oscillatory shear remains, suggesting that reproductive aging plays a role in the changes observed in conduit artery shear patterns across the menopause transition. In our discussion of findings, we suggest that reduced NO bioavailability may play a role in the observed increases to retrograde and oscillatory shear with reproductive aging. However, we did not directly measure NO in women and acknowledge this as a limitation to our study.

In conclusion, this work contributes to the growing body of literature identifying the unique accelerated rise in CVD risk factors that take place during the menopause transition, placing postmenopausal women at heightened risk for CVD development. Our contribution to this area is the finding that arterial shear stress changes during menopause to adopt a greater oscillatory pattern that is well known to be atherogenic. Further examination is necessary to fully understand these observations in order to test strategies on women aimed at attenuating the vascular dysfunction associated with retrograde shear stress.

## Conflict of Interest

None declared.
